# Two Imported Chikungunya Cases, Taiwan

**DOI:** 10.3201/eid1408.071304

**Published:** 2008-08

**Authors:** Pei-Yun Shu, Cheng-Fen Yang, Chien-Ling Su, Chung-Yu Chen, Shu-Fen Chang, Kun-Hsien Tsai, Chia-Hsin Cheng, Jyh-Hsiung Huang

**Affiliations:** * Centers for Disease Control, Taipei, Taiwan, Republic of China

**Keywords:** chikungunya, chikungunya virus, fever screening, Taiwan, RT-PCR, phylogenetic analysis, letter

**To the Editor:** Chikungunya is a reemerging infectious disease, endemic to Africa and Southeast Asia, caused by a mosquito-borne alphavirus in the family *Togaviridae*. Numerous chikungunya outbreaks have been reported in Africa and Southeast Asia since chikungunya virus (CHIKV) was first isolated in Tanzania in 1953 (*1*). Since 2005, several Indian Ocean islands and India have experienced massive CHIKV outbreaks caused by the East/Central/South African genotype (*2*,*3*), whereas all earlier isolates from India during 1963–1973 were of the Asian genotype (*4*). Other chikungunya outbreaks caused by the Asian genotype were frequently reported during 1960–2003 in many Southeast Asian countries, including India, Malaysia, Indonesia, Cambodia, Vietnam, Myanmar, Pakistan, the Philippines, and Thailand. Epidemics caused by reemerging CHIKV were reported in Indonesia and Malaysia during 2005–2007 (*1*,*5*).

We have previously reported on fever screening at airports in Taiwan as part of active surveillance for a panel of notifiable infectious diseases such as dengue, gastroenteritis caused by enteric bacteria, malaria, and yellow fever (*6*). The activity is carried out by using infrared thermal scanners to measure the body temperature of arriving passengers. Diagnostic testing algorithms for patients being screened for fever were based on evaluation by airport clinicians. The rationale behind this process is to minimize local outbreaks by reducing the number of imported cases. We report 2 imported chikungunya case-patients identified in Taiwan by fever screening at airports; 1 had returned from Singapore in 2006, infected with CHIKV East/Central/South African genotype, and the other had returned from Indonesia in 2007, infected with the Asian genotype.

To assess viremic fever patients with alphavirus infection, a multiplex 1-step SYBR Green I-based real-time reverse transcription–PCR (RT-PCR) was developed. A cocktail consisting of 3 sets of primers was mixed and used for RT-PCR screening. The alphavirus-specific primer set (AL-2: 5′-AAG CTY CGC GTC CTT TAC CAA AG-3′ and AL-3: 5′-GTG GTG TCA AAC CCT ATC CA-3′) targeted a consensus region of the nonstructural protein 1 (*nsp1*) genes to detect all alphaviruses. The CHIKV-specific primer set (F-CHIK: 5′-AAG CTY CGC GTC CTT TAC CAA AG-3′ and R-CHIK: 5′-CCA AAT TGT CCY GGT CTT CCT-3′) targeted a region of the envelope protein 1 (E1) gene of CHIKVs (*7*). The Ross River virus–specific primer set (RRV-1: 5′-GGG TAG AGA GAA GTT YGT GGT YAG-3′ and RRV-2: 5′-CGG TAT ATC TGG YGG TGT RTG C-3′) targeted a region of the envelope protein 2 (E2) gene of Ross River virus. Positive results were then confirmed by gene sequence analysis, virus isolation, and serologic tests. The nucleotide sequences of complete structural polyprotein genes were determined as previously described and submitted to GenBank (accession nos. EU192142 and EU192143) (*3*,*8*). A phylogenetic tree, based on a total of 23 CHIKV partial E1 gene sequences (255 bp), was drawn to trace the origin of 2 CHIKV isolates reported in this study (Figure).

**Figure:**
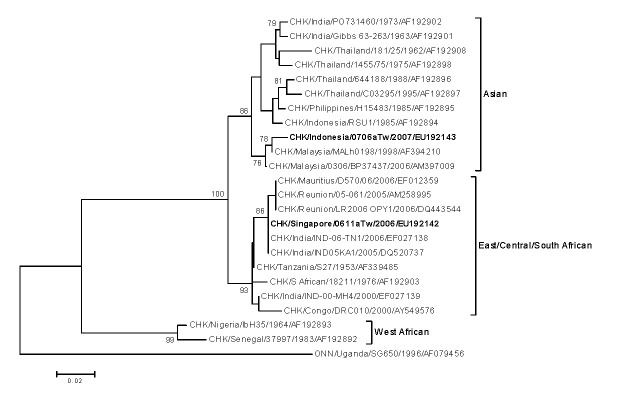
Phylogenetic relationships of chikungunya virus (CHIKV) isolates from 2 imported cases in Taiwan. The tree was constructed by the neighbor-joining method using partial nucleotide sequences of envelope protein 1 (E1) gene (255 bp) of 23 CHIKV strains. O’nyong-nyong (ONN) virus sequence was used as the outgroup virus. Bootstrap support values >75 are shown. The 2 imported CHIKV strains in Taiwan are designated by **boldface** type. Viruses were identified by using the nomenclature of virus/country/strain/year of isolation/GenBank accession no. Scale bar indicates substitutions per site.

The initial imported chikungunya case was detected at Taiwan Taoyuan International Airport on November, 20, 2006, in a 13-year-old Taiwanese boy who was returning from studying at an international educational training center in Singapore. The second imported case was also detected at Taiwan Taoyuan International Airport on June, 20, 2007, in a 5-year-old boy on his return from visiting relatives in East Kalimantan Province, Indonesia, with his mother. Both case-patients had fever, fatigue, generalized arthralgia, and rash. Real-time RT-PCR screening showed a high level of alphavirus, but not flavivirus, viremia on day 2 (Singapore imported case) and day 3 (Indonesia imported case) acute-phase samples. Serodiagnosis with immunofluorescent antibody assay (immunoglobulin M + G + A titers >640), and ELISA showed positive seroconversions for both patients.

Analysis showed that these 2 imported cases were introduced from Singapore and Indonesia and that the patients were infected with CHIKV of East/Central/South African genotype and Asian genotype, respectively. Unlike dengue, chikungunya is not endemic to Singapore. However, a small chikungunya outbreak caused by an Indian strain of East/Central/South African genotype transmitted by *Aedes aegypti* was reported in January 2008; this occurrence suggests that imported CHIKV may not be detected because of limited transmission and because the signs and symptoms may be mistaken for those of dengue (*9*). In contrast, chikungunya has been endemic to Indonesia since 1973. Indonesia had epidemic outbreaks in 1980, 1983–1984, and yearly outbreaks after 1998. In following the ongoing chikungunya epidemic, we have identified 4 additional imported chikungunya cases from Indonesia since July 2007.

A recent chikungunya outbreak in Italy demonstrated that *Aedes albopictus* is a competent vector that can initiate local transmission of imported CHIKV (*10*). In Taiwan, *Ae. albopictus* is distributed throughout the island, and *Ae. aegypti* is distributed only in southern Taiwan. With increasing numbers of imported CHIKV infections, the risk for local transmission is similar to that of dengue, especially in southern Taiwan. Our results show that CHIKVs of both genotypes are spreading in Southeast Asia. The cocirculation of dengue and chikungunya would likely be increased in many Southeast Asian and African countries because of the rise in international travel and the wide distribution of the competent vectors, *Ae. albopictus* and *Ae. aegypti*.
